# Detailed findings of videofluoroscopic examination among patients with Parkinson's disease on the effect of cervical percutaneous interferential current stimulation

**DOI:** 10.3389/fneur.2023.1279161

**Published:** 2023-11-07

**Authors:** Masahiro Nakamori, Megumi Toko, Hidetada Yamada, Yuki Hayashi, Azusa Haruta, Aya Hiraoka, Mineka Yoshikawa, Toshikazu Nagasaki, Kai Ushio, Kohei Yoshikawa, Yoshitaka Shimizu, Yukio Mikami, Hirofumi Maruyama

**Affiliations:** ^1^Department of Clinical Neuroscience and Therapeutics, Hiroshima University Graduate School of Biomedical and Health Sciences, Hiroshima, Japan; ^2^Department of Advanced Prosthodontics, Hiroshima University Graduate School of Biomedical and Health Sciences, Hiroshima, Japan; ^3^Department of Oral and Maxillofacial Radiology, Hiroshima University Graduate School of Biomedical and Health Sciences, Hiroshima, Japan; ^4^Department of Rehabilitation Medicine, Hiroshima University Hospital, Hiroshima, Japan; ^5^Department of Dental Anesthesiology, Hiroshima University Graduate School of Biomedical and Health Sciences, Hiroshima, Japan

**Keywords:** Parkinson's disease, dysphagia, interferential current sensory stimulation, videofluoroscopic examination, temporal analysis

## Abstract

**Introduction:**

Parkinson's disease (PD) leads to various types of swallowing disorders. We investigated the effect of cervical percutaneous interferential current stimulation on dysphagia. By conducting detailed qualitative and quantitative analysis of videofluoroscopic examination, we aimed to understand dysphagia in patients with PD and investigate its effects on swallowing function.

**Methods:**

Patients received cervical percutaneous interferential current stimulation for 20 min twice a week for 8 weeks. In this exploratory study, we evaluated aspiration/laryngeal penetration, oral cavity residue, vallecular residue, and pharyngeal residue. In addition, we performed temporal analysis.

**Results:**

Twenty-five patients were completely evaluated. At baseline, the proportions of laryngeal penetration/aspiration, oral cavity residue, epiglottic vallecula residue, and pharyngeal residue were 40.0, 88.0, 72.0, 60.0, and 16.0%, respectively. Conversely, pharyngeal transit time, laryngeal elevation delay time, pharyngeal delay time, and swallowing reflex delay were nearly within the normal ranges. Cervical percutaneous interferential current sensory stimulation improved only oral cavity residue at the end of the intervention, from 88.0 to 56.0%.

**Discussion:**

Patients with PD demonstrated remarkably high frequencies of residues in the oral and pharyngeal regions. The usefulness of cervical interferential current stimulation was partially demonstrated for oral cavity residue. Considering that PD exhibits diverse symptoms, further accumulation of cases and knowledge is warranted.

**Trial registration:**

jRCTs062220013.

## 1. Introduction

Neurological disorders often accompany dysphagia, and dysphagia in patients with Parkinson's disease (PD) holds significant importance. Aspiration pneumonia, arising from dysphagia, represents a major cause of mortality among patients with PD, highlighting the unmet medical need in managing swallowing difficulties in this population. PD leads to the following types of swallowing disorders: abnormal transport from the oral cavity and pharynx (1), delayed swallowing reflex (2), pharynx residue (3), and others. In addition, silent aspiration, which is caused by decreased sensation in the pharynx and larynx, is also serious and characteristic in patients with PD (3).

We planned an intervention trial that primarily focused on silent aspiration, aiming to activate the sensory nerves through cervical percutaneous interferential current stimulation. Recent innovations in neurological stimulation methods include cervical percutaneous electrical stimulation to enhance neuromuscular function. Pulsed current approaches show promise in inducing muscle contractions to treat dysphagia but are associated with discomfort (4). An alternative method, interferential current sensory stimulation, activates peripheral nerves in the pharynx and larynx to heighten sensitivity and protect the airway (5). Reports suggest that electrical stimulation devices can enhance swallowing without muscle contraction (6, 7). Interferential currents penetrate deeper tissues comfortably compared to pulsed currents, holding potential for alleviating dysphagia (8). Studies have highlighted enhanced saliva production (9), reduced pharyngeal latency, increased swallowing frequency, and improved airway sensitivity (8). Cervical percutaneous interferential current stimulation might benefit patients with dysphagia by enhancing airway defense and nutrition (10). Moreover, cervical percutaneous interferential current can stimulate the central pattern generator (CPG) and improve the swallowing reflex (11). Previously, we investigated the relationship between videofluoroscopic examination (VF) and brain lesion sites in patients with stroke and reported a correlation between delayed swallowing reflex initiation and basal ganglia lesions (12). PD also involves abnormalities in the cerebral basal ganglia network. Therefore, the effectiveness of cervical percutaneous interferential current stimulation for the swallowing reflex might be considered.

In this study, the main objective was to assess the improvement of cough reflex in patients with PD. However, the complexity of dysphagia in PD arises from various factors, necessitating a comprehensive grasp of swallowing dynamics. We focused on exploring how cervical percutaneous interferential current stimulation impacts swallowing function in patients with PD. Through qualitative and quantitative analysis of VF, which is the gold standard method for evaluation, this study aimed to enhance the understanding of PD-related dysphagia and thoroughly examine the effects of cervical percutaneous interferential current stimulation.

## 2. Material and methods

### 2.1. Ethics approval, registration, and patient consent

This research received authorization from the Certified Review Board at Hiroshima University (<city>Hiroshima </city>, Japan) (approval ID: CRB6180006) and adhered to the directives of the federal administration in line with the principles outlined in the 1964 Declaration of Helsinki. It has been duly recorded in the jRCT database (jRCTs062220013). Comprehensive written consent was acquired from all participants involved in the study.

### 2.2. Study design and protocol

The study's design and protocol were previously published (13). The methodology consisted of a single-arm, open-label study that adhered to the reporting guidelines outlined in SPIRIT (14). Our investigation centered on assessing the effectiveness and safety of percutaneous neck interferential current stimulation in patients diagnosed with PD, as per the criteria set by the Movement Disorder Society, falling within Hoehn-Yahr stages 2–4 (15). The study was conducted at Hiroshima University Hospital.

We enrolled individuals who met the criteria of clinically probable or established PD as defined by the Movement Disorder Society criteria, with Hoehn-Yahr stages 2–4 at the time of registration. Additionally, participants needed to be capable of visiting the hospital twice weekly and provide informed written consent. Eligibility was restricted to patients aged between 19 and 86 years whose levodopa dosage had remained constant for over a month. Those with implanted pacemakers or defibrillators, undergoing deep brain stimulation, pregnant or attempting to conceive, diagnosed with or having a history of head or neck cancer, currently experiencing active pneumonia, or possessing a history of swallowing rehabilitation, were excluded from the study.

**Figure 1:**
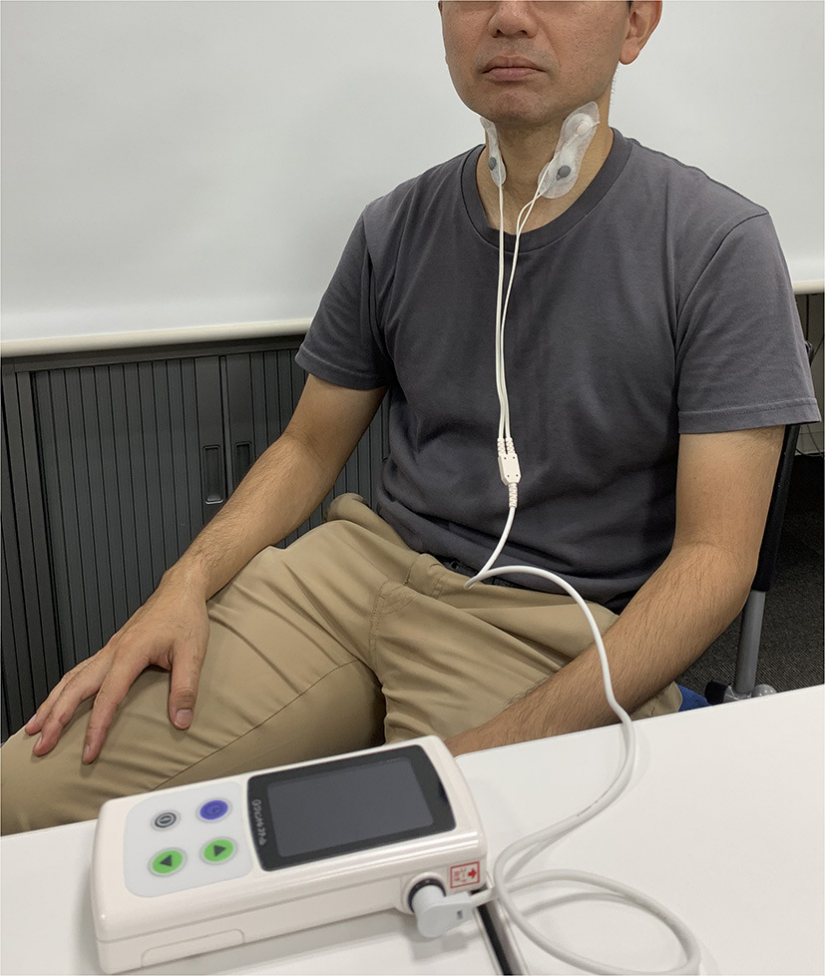
Picture of cervical percutaneous interferential current stimulation.

Participants underwent cervical interferential current stimulation for 20 min, twice a week, over an 8-week period. The stimulation was administered using a Gentle Stim^®^ device from FoodCare Co., Ltd., Kanagawa, Japan. Electrode pads were applied to the front of the neck to stimulate the swallowing-related (glossopharyngeal nerve and superior laryngeal) nerves. A 50 Hz swallowing reflex interferential current stimulation was utilized because of its lower threshold in comparison to pulse stimulation, resulting in minimal sensation for patients. Stimulation adhered to a standardized protocol, with the maximum stimulation current set below the threshold at which the patient could perceive electrical sensations, ranging from 2.0 to 2.5 mA. Stimulation was administered consistently and repeatedly. Figure 1 shows the landscape of the stimuli.

Evaluations, except for VF, were conducted every 4 weeks from the start of the intervention to 16 weeks post-intervention initiation. VF assessments were conducted every 8 weeks within the same timeframe to minimize the radiation exposure.

The primary and secondary endpoints were outlined in a prior publication (13). As part of this study, we conducted a thorough and detailed evaluation using VF, a recognized gold standard method for evaluating swallowing function. Our study concentrated specifically on VF findings.

### 2.3. Videofluoroscopic examination

An X-ray imaging system (Ultimax-i, CANON MEDICAL SYSTEM CORPORATION, Tochigi, Japan) was used and the tests were performed with patients in a seated position. The test material was 3 mL of water with 30%/w barium contrast medium (Barytester A240 Powder^®^, FUSHIMI Pharmaceutical Co. Ltd, Kagawa, Japan), which the patients were instructed to swallow after it was delivered via a syringe to the floor of the mouth. The evaluation using 3 mL of water is relatively widely accepted and implemented for assessing swallowing function (16). In addition, 3 mL should always be used in evaluations to prioritize safety in individuals with swallowing disorders (17). Another evaluation method, the simple water drinking test known as the Modified Water Swallow Test (18), also uses this volume of water. Therefore, the present study also used 3 mL of water. The imaging with the X-ray system was performed forward toward the lips, back to the pharyngeal wall, up to the nasal cavity, and downward to the upper esophageal sphincter, obtaining a side VF recording of 30 frames per second. The data were recorded on a DVD. Three blinded dentists (A Hiraoka, A Haruta, and MY) with specialized experience in evaluating videofluorographic recordings and established protocols following training on VF assessment determined the presence or absence of laryngeal penetration/aspiration, and clearance or prevalence of oral cavity residue, vallecular residue, or pharyngeal residue after one swallow. We also performed a semi-quantitative evaluation of oral cavity, vallecular, and pharyngeal residues, which were scored as grade 0 (no residue), grade 1 (thin coating of residue), or grade 2 (obvious residue). Aspiration/laryngeal penetration was categorized as grade 0 (none), grade 1 (laryngeal penetration), or grade 2 (aspiration beyond the vocal cords). In addition, the passage time of each anatomical landmark was measured and temporal analysis was performed. We calculated pharyngeal transit time (PTT), laryngeal elevation delay time (LEDT), and pharyngeal delay time (PDT). PTT is defined as the time from when the bolus tip reaches the lower border of the mandible to the complete passage of the bolus tail through the esophageal inlet (normal range 0.43–1.11 s) (19). LEDT is defined as the time from when the bolus tip reaches the vallecula to the peak of laryngeal elevation, in which the favorable cut-off value is 0.32 s (20). PDT is defined as the time from when the bolus tip reaches the intersection of the lower border of the mandible and the base of the tongue to the initiation of laryngeal elevation, in which the duration among healthy adults is 0–0.2 s (21). Furthermore, we evaluated the presence or absence of swallowing reflex delay, defined as liquid remaining in the pyriform sinuses for >0.1 s (3 frames) before swallowing (12). Three observers discussed their observations and reached a consensus for each observation or measurement.

### 2.4. Data acquisition

Clinical evaluation and diagnosis were conducted by two neurologists (MN and HY). The recorded data included body mass index, grip power, calf circumference, disease duration, alcohol drinking and smoking habits, Unified Parkinson's Disease Rating Scale score (22), medication, and Functional Oral Intake Scale score (23). Tongue pressure was assessed as previously described (24, 25). The levodopa equivalent daily dose (LEDD) was calculated based on a recent study (26). All evaluations were conducted in the ON state.

### 2.5. Sample size

We determined the necessary sample size based on initial assessments of coughing in individuals with neurodegenerative conditions. In these assessments, 28.6% of individuals exhibited a normal cough reflex following a 1% citric acid challenge. Assuming that 50% of individuals would demonstrate a normal cough reflex after 8 weeks of treatment, the estimated sample size was 27 participants. This calculation was made using an alpha level of 0.10, a power of 0.80, and accounting for a dropout rate of 10%.

### 2.6. Statistical analysis

The data are expressed as means ± standard deviation or medians (minimum, maximum) for continuous variables and as frequencies and percentages for discrete variables. Statistical analysis was performed using JMP statistical software, version 16 (SAS Institute Inc., Cary, NC, USA). To assess the efficacy of cervical percutaneous interferential current stimulation, we compared the VF results for each patient before the initial intervention with those obtained 8 weeks after the start or 8 weeks after the conclusion of the intervention (16 weeks from initiation). Additionally, we conducted a statistical comparison between the group that exhibited improvement and the group that did not. For the assessment of intergroup variances, appropriate statistical tests such as the χ2 test, Mann–Whitney *U*-test, or unpaired *t*-test were employed. The baseline data of patients were analyzed, and two-step strategies were applied to evaluate the relative importance of variables that exhibited improvement of VF findings using multiple logistic analysis. First, univariate analysis was performed. Subsequently, a multi-factorial analysis was performed with selected factors with a *p*-value <0.05 in univariate analysis. The correlation between factors was calculated using Pearson's correlation coefficients. We used basic factors (age, UPDRS total score tongue pressure). Statistical significance was set at *p* < 0.05.

## 3. Results

In this study, 27 participants were enrolled, and within 4 weeks after the start, two individuals withdrew their consent because of personal reasons. As a result, intervention and evaluation were conducted with 25 participants. The intervention was conducted without any deviations.

The patient demographics and swallowing-related indicator data at baseline (pre-intervention) are shown in Table 1. The laryngeal penetration/aspiration, oral cavity residue, epiglottic vallecula residue, and pharyngeal residue were found in remarkable frequency. Conversely, PTT, LEDT, and PDT were almost within the normal range. The number of patients who exhibited deviations from the standard values of LEDT and PDT delays was three and one, respectively. The number of swallowing reflex delays was 4 (16.0%).

**Table 1:** Patient backgrounds and indicators related to swallowing function.

	***n* = 25**
Age, years	72.0 ± 5.9
Sex (female), *n* (%)	9 (36.0)
Duration, years	6 (1, 20)
Body mass index, kg/m^2^	21.2 ± 2.8
Alcohol consumption, *n* (%)	2 (8.0)
Current smoking, *n* (%)	3 (12.0)
Hoehn & Yahr stage	3 (2, 4)
UPDRS score (total)	37 (19, 76)
UPDRS score (part 3)	23 (10, 50)
Dopa, mg	360 ± 203
LEDD, mg	583 ± 395
Maximum handgrip strength, kg	24.8 ± 5.6
Calf circumference, cm	33.8 ± 3.3
FOIS	7 (6, 7)
Tongue pressure, kPa	30.6 ± 8.5
**VF findings**	
Laryngeal penetration or Aspiration, *n* (%)	10 (40.0)
Oral cavity residue, *n* (%)	22 (88.0)
Epiglottic vallecula residue, *n* (%)	18 (72.0)
Pharyngeal residue, *n* (%)	15 (60.0)
Swallowing reflex delay, *n* (%)	4 (16.0)
Pharyngeal transit time, second	0.719 ± 0.122
Laryngeal elevation delay time, second	0.217 ± 0.146
Pharyngeal delay time, second	0.002 ± 0.134

UPDRS, Unified Parkinson's Disease Rating Scale; LEDD, Levodopa equivalent daily dose; FOIS, Functional Oral Intake Scale; VF, videofluoroscopic examination.

Data are expressed as mean ± standard deviation or median (minimum, maximum) for continuous variables and as frequencies and percentages for discrete variables.

**Figure 2:**
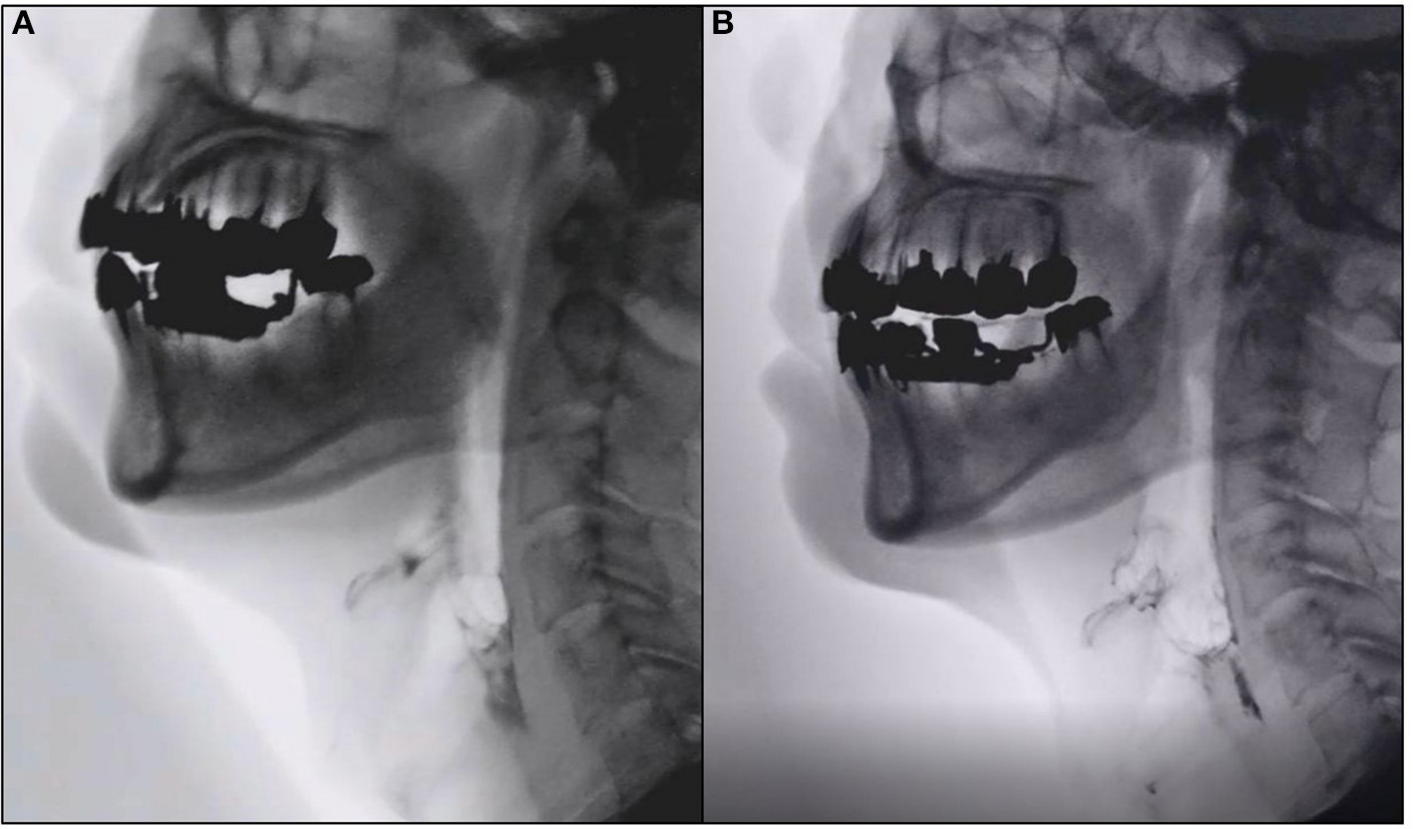
Representative images of videofluoroscopic examination. Images of videofluoroscopic examination from Case 5. **(A)** Pre-intervention (0 weeks). **(B)** Eight weeks post-intervention showing marked improvement in oral cavity residue.

The transitions of indicators at baseline, at the end of the intervention (8 weeks from the initiation of intervention), and at 8 weeks after the last intervention (16 weeks from the initiation of intervention) are shown in Table 2. At the intervention endpoint (8 weeks), oral cavity residue showed a significant improvement compared to that before the intervention (Figure 2). However, at 8 weeks after the intervention ended (16 weeks), no significant sustained improvement was observed. Additionally, no significant changes were observed in laryngeal penetration/aspiration, vallecular residue, and pharyngeal residue throughout the entire course when compared to the baseline. Moreover, no significant changes were found in PTT, LEDT, and PDT during the temporal analysis. Similarly, no significant change was observed in swallowing reflex delay. A semi-quantitative evaluation of aspiration/laryngeal penetration and oral cavity, vallecular, and pharyngeal residues was also performed, which demonstrated a significant improvement in the oral cavity residue at the intervention endpoint (8 weeks). However, no significant changes were observed in aspiration/laryngeal penetration or vallecular and pharyngeal residues (Supplementary Table 1).

**Table 2:** Transition of indicators at baseline, 8, and 16 weeks from the start of the intervention.

	**0 weeks (pre-intervention)**	**8 weeks (post-intervention)**	***p-*value**	**16 weeks**	***p-*value**
Body mass index, kg/m^2^	21.2 ± 2.8	21.3 ± 2.8	0.899	21.0 ± 2.5	0.782
UPDRS (total)	37 (19, 76)	42 (14, 78)	0.853	44 (15, 80)	0.554
UPDRS (part 3)	23 (10, 50)	25 (10, 51)	0.930	28 (11, 51)	0.669
Dopa, mg	360 ± 203	360 ± 203	1.000	360 ± 203	1.000
LEDD, mg	583 ± 395	583 ± 395	1.000	589 ± 392	0.957
Maximum handgrip strength, kg	24.8 ± 5.6	25.5 ± 5.4	0.665	26.7 ± 6.4	0.276
Calf circumference, cm	33.8 ± 3.3	33.8 ± 3.5	1.000	34.0 ± 3.2	0.830
FOIS	7 (6, 7)	7 (6, 7)	1.000	7 (6, 7)	1.000
Tongue pressure, kPa	30.6 ± 8.5	33.6 ± 8.0	0.199	34.6 ± 8.4	0.101
**VF findings**					
Laryngeal penetration or aspiration, *n* (%)	10 (40.0)	9 (36.0)	0.771	11 (44.0)	0.775
Oral cavity residue, *n* (%)	22 (88.0)	14 (56.0)	0.012^*^	19 (76.0)	0.270
Epiglottic vallecula residue, *n* (%)	18 (72.0)	17 (68.0)	0.758	15 (60.0)	0.371
Pharyngeal residue, *n* (%)	15 (60.0)	11 (44.0)	0.258	14 (56.0)	0.775
Swallowing reflex delay, *n* (%)	4 (16.0)	3 (12.0)	0.684	5 (20.0)	0.713
Pharyngeal transit time, s	0.719 ± 0.122	0.750 ± 0.167	0.461	0.755 ± 0.183	0.411
Laryngeal elevation delay time, s	0.217 ± 0.146	0.215 ± 0.189	0.967	0.256 ± 0.117	0.301
Pharyngeal delay time, s	0.002 ± 0.134	−0.020 ± 0.193	0.645	0.049 ± 0.142	0.238

UPDRS, Unified Parkinson's Disease Rating Scale; LEDD, Levodopa equivalent daily dose; FOIS, Functional Oral Intake Scale; EAT-10, Eating Assessment Tool-10; VF, videofluoroscopic examination.

Data are expressed as mean ± standard deviation or median (minimum, maximum) for continuous variables, and frequencies and percentages for discrete variables. Univariate analyses were performed compared to the baseline (0 weeks). ^*^p < 0.05.

At the baseline stage, there were 22 participants with oral cavity residue, and at the end of the 8-week intervention, nine patients showed improvement. Therefore, using the baseline data, a univariate analysis was conducted to examine the factors associated with improvement between these nine individuals and 13 who did not exhibit improvement. As a result, a higher body mass index and higher calf circumference were significantly associated with improved oral cavity residue (*p* < 0.05) (Table 3). These factors showed a strong correlation in Pearson correlation analysis, with a correlation coefficient of 0.782 (*p* < 0.001). Therefore, we conducted multivariate analysis by including age (Model 1), UPDRS total score (Model 2), and tongue pressure (Model 3), respectively, for each of body mass index and calf circumference to determine their validity as factors related to improving oral cavity residue. The results showed that body mass index and calf circumference were both significant correlating factors (Table 4). Conversely, three patients had no oral cavity residue at baseline, although one individual did at the end of the 8-week intervention. The disease duration, body mass index, calf circumference, and LEDD of the patient was 10 years, 17.5 kg/m^2^, 32 cm, and 1,300 mg, respectively.

**Table 3:** Comparison between patients with and without improvement and non-improvement in oral cavity residue.

	**Improved (*n* = 9)**	**Not improved (*n* = 13)**	***p-*value**
Age, years	71.1 ± 4.3	73.1 ± 7.4	0.481
Sex (female), *n* (%)	3 (33.3)	4 (30.8)	0.899
Duration, years	6 (3, 20)	10 (1, 13)	0.788
Body mass index, kg/m^2^	23.9 ± 2.2	19.6 ± 1.8	< 0.001^*^
Alcohol consumption, *n* (%)	0 (0)	1 (7.7)	0.394
Current smoking, *n* (%)	1 (11.1)	2 (15.4)	0.774
Hoehn & Yahr stage	3 (2, 4)	3 (2, 3)	0.616
UPDRS score (total)	36 (24, 76)	35 (19, 76)	0.738
UPDRS score (part 3)	23 (10, 50)	22 (14, 48)	0.920
Dopa, mg	356 ± 116	327 ± 219	0.724
LEDD, mg	587 ± 393	533 ± 373	0.745
Maximum handgrip strength, kg	27.3 ± 5.7	23.7 ± 5.5	0.148
Calf circumference, cm	36.3 ± 2.5	32.1 ± 3.1	0.003^*^
FOIS	7 (7, 7)	7 (6, 7)	0.460
Tongue pressure, kPa	32.7 ± 9.5	28.5 ± 6.7	0.237

UPDRS, Unified Parkinson's Disease Rating Scale; LEDD, Levodopa equivalent daily dose; FOIS, Functional Oral Intake Scale; EAT-10, Eating Assessment Tool-10; VF, videofluoroscopic examination.

Data are expressed as mean ± standard deviation or median (minimum, maximum) for continuous variables, and frequencies and percentages for discrete variables. ^*^p < 0.05.

**Table 4:** Multivariate analysis of oral cavity residue improvement.

	**Model 1**	***p-*value**	**Model 2**	***p-*value**	**Model 3**	***p-*value**
	**Odds ratio (95% CI)**		**Odds ratio (95% CI)**		**Odds ratio (95% CI)**	
Body mass index	3.97 (1.16–13.54)	0.028^*^	3.83 (1.14–12.85)	0.030^*^	3.97 (1.16–13.58)	0.028^*^
Calf circumference	1.75 (1.12–2.74)	0.014^*^	1.73 (1.09–2.75)	0.020^*^	1.71 (1.17–3.05)	0.022^*^

CI, confidence interval.

Body mass index and calf circumference were identified as factors with *p* < 0.05 in univariate analyses for improvement in oral cavity residue. Multivariate analyses were performed using each factor and the basic factors; age (Model 1), Unified Parkinson's Disease Rating Scale total score (Model 2), and tongue pressure (Model 3), respectively.

^*^p < 0.05.

## 4. Discussion

In this study, we extensively investigated the swallowing disorders in patients with PD using the gold standard VF. We have previously conducted detailed examinations of swallowing disorders in patients with amyotrophic lateral sclerosis and stroke using similar methods (24, 27, 28). These diseases are generally neurologic disorders characterized by paralysis and muscle weakness. Additionally, a decrease in tongue pressure and oral phase impairments are the central aspects of swallowing disorders, with exceptions such as Wallenberg's syndrome (29). PD, on the other hand, primarily manifests as bradykinesia, without typical paralysis. Extrapolating knowledge from other neurological disorders to evaluate swallowing disorders in PD is impractical. Furthermore, our study results suggested that diverse factors contribute to swallowing disorders in patients with PD.

Laryngeal penetration/aspiration, oral cavity residue, epiglottic vallecula residue, and pharyngeal residue were observed at a significant frequency. Tongue pressure was well-maintained. Therefore, muscle weakness was probably not the cause. The lack of efficient motion is due to bradykinesia and muscle rigidity. Conversely, the temporal analysis of the swallowing reflex showed that it was mostly within the normal range. When comparing VF and brain lesion sites in patients with stroke, there is a correlation between delayed swallowing reflex initiation and basal ganglia lesions (12, 20). Considering that PD also involves abnormalities in the cerebral basal ganglia network, we anticipated the possibility of delayed swallowing reflex initiation. However, the results contradicted the expectations. Neurodegenerative diseases such as PD, in contrast to stroke, involve systematic disruptions in the nervous system, leading to distinct clinical manifestations. Moreover, the CPG for swallowing is located in the medulla near the nucleus ambiguous and solitary tract nucleus (30, 31). The onset of PD is associated with the dorsal motor nucleus of the vagus nerve, which is in a different location. This suggests that CPG impairment might be bypassed in PD.

In this study, cervical percutaneous interferential current stimulation significantly improved oral cavity residue. We speculate that sensory stimulation through Gentle Stim^®^–which assumes the stimulation of the glossopharyngeal and superior laryngeal nerves—may have activated the sensation and facilitated oral-phase initiation, as the posterior one-third of the tongue is controlled by the glossopharyngeal nerve. Furthermore, high body mass index and calf circumference were associated with improvement in oral cavity residue. Previous studies in healthy older individuals reported the positive correlation of body mass index and calf circumference with tongue pressure and tongue thickness (32). A thicker tongue reduces oral cavity volume and makes it more likely for tongue pressure to increase. A decrease in tongue pressure and tongue thickness has been associated with a decline in oral phase, namely, the passage of bolus from the oral cavity to the pharynx (24, 27). Based on these previous reports, it is consistent that body mass index and calf circumference are also involved in oral residue in patients with PD. Therefore, patients with maintained physical stature and muscle mass may potentially benefit more from cervical interferential current stimulation. However, further replication and investigation are essential to elucidate these factors conclusively in the context.

In addition to the cervical percutaneous interferential current stimulation used in this study, low-frequency neuromuscular electrical stimulation has also been reported to be a useful treatment for dysphagia (33). Low-frequency neuromuscular electrical stimulation primarily aims to induce muscle contractions, which can potentially contribute to the improvement of the muscle strength of swallowing-related muscles. In contrast, the interferential current stimulation used in the present study provides stimulation at levels that do not induce muscle contractions. Rather, this stimulation is characterized by interference waves reaching deep tissues and activating sensory nerves, resulting in less pain or discomfort caused by muscle contractions. As muscle strength, including tongue pressure, is relatively preserved in PD, it is important to select stimulation methods based on the pathophysiology of swallowing disorders. Future studies are needed to investigate how low-frequency neuromuscular electrical stimulation contributes to swallowing disorders in PD.

This study has several limitations. First, this study is a single-site single-group intervention trial. A randomized controlled trial including a non-intervention/sham stimulation group for intergroup comparison should be considered in future research. This study is the first investigation into the effectiveness of cervical interferential current stimulation among patients with PD. One of the objectives of this exploratory study was to explore factors that show improvement through the intervention of percutaneous interferential current stimulation. In the future, despite ethical challenges, intergroup comparison trials should be conducted. Second, one of the challenges of this exploratory study was the limited number of patients under investigation. This study focused on cervical interferential current stimulation as the primary endpoint to improve cough reflex testing. Therefore, the sample size was determined based on previous studies and existing literature. However, to comprehensively examine and analyze swallowing disorders in patients with PD and diverse symptoms, a substantial number of patients must be included for investigation. Therefore, further recruitment of patients is necessary for future research.

This exploratory study provided new insights into swallowing disorders in patients with PD. Additionally, the usefulness of cervical interferential current stimulation was partially demonstrated. Considering that PD causes diverse symptoms, further recruitment of patients and knowledge is warranted.

## Data availability statement

The raw data supporting the conclusions of this article will be made available by the authors, without undue reservation.

## Ethics statement

The studies involving humans were approved by Certified Review Board at Hiroshima University. The studies were conducted in accordance with the local legislation and institutional requirements. The participants provided their written informed consent to participate in this study.

## Author contributions

MN: Conceptualization, Data curation, Formal analysis, Funding acquisition, Investigation, Methodology, Project administration, Validation, Writing—original draft. MT: Conceptualization, Data curation, Investigation, Methodology, Validation, Writing—original draft. HY: Conceptualization, Data curation, Formal analysis, Investigation, Methodology, Validation, Writing—original draft. YH: Data curation, Writing—original draft. AHa: Formal analysis, Investigation, Methodology, Writing—original draft. AHi: Formal analysis, Investigation, Methodology, Writing—original draft. MY: Investigation, Methodology, Validation, Writing—original draft. TN: Data curation, Investigation, Methodology, Writing—review & editing. KU: Conceptualization, Methodology, Writing—review & editing. KY: Data curation, Investigation, Methodology, Writing—original draft. YS: Conceptualization, Methodology, Writing—review & editing. YM: Project administration, Supervision, Validation, Writing—review & editing. HM: Conceptualization, Supervision, Validation, Writing—review & editing.
