# Supplementary material for: Detailed findings of videofluoroscopic examination among patients with Parkinson's disease on the effect of cervical percutaneous interferential current stimulation
Source: Front Neurol. 2023 Nov 7;14:1279161. doi: 10.3389/fneur.2023.1279161 (PMC10664245; doi:10.3389/fneur.2023.1279161)
Supplement: Supplementary file 1 [file Table_1.docx]

Supplementary Material

**Supplemental Table 1. Transition in semi-quantitative evaluations of aspiration/laryngeal penetration and oral cavity, vallecular, and pharyngeal residues at baseline, 8, and 16 weeks from the start of the intervention**

|  | 0 weeks  (pre-intervention) | 8 weeks  (post-intervention) | p value | 16 weeks | p value |
| --- | --- | --- | --- | --- | --- |
| Laryngeal penetration or aspiration | 0.48±0.65 | 0.40±0.58 | 0.648 | 0.52±0.65 | 0.830 |
| Oral cavity residue | 1.12±0.60 | 0.64±0.64 | 0.009 | 1.00±0.71 | 0.521 |
| Epiglottic vallecula residue | 0.92±0.70 | 0.92±0.76 | 1.000 | 0.96±0.89 | 0.861 |
| Pharyngeal residue | 0.76±0.72 | 0.64±0.81 | 0.583 | 0.76±0.78 | 1.000 |

Data are expressed as mean ± standard deviation.

Univariate analyses were performed compared to baseline (0 weeks). *p<0.05.
